# Somatic cell count in dairy goats I: association with infectious and non-infectious factors

**DOI:** 10.1186/s12917-024-04348-6

**Published:** 2024-11-06

**Authors:** Marit Smistad, Ragnhild Aabøe Inglingstad, Liv Sølverød, Siv Skeie, Bjørn Gunnar Hansen

**Affiliations:** 1 R&D Department, Farm Advisory Services, TINE SA, BTB-NMBU, Pb. 5003, Ås, 1432 Norway; 2 TINE Mastitis Laboratory, Pb 2039, Molde, 6402 Norway; 3 Faculty of Chemistry, Biotechnology and Food Science, Norwegian University of Life Sciences, P.O Box 5003, Ås, N-1432 Norway

**Keywords:** *S. aureus*, Udder health, Season, Milk quality

## Abstract

**Background:**

Intramammary infections negatively affect milk quality, animal welfare and productivity in the dairy industry. Somatic cell count (SCC) is the most used screening tool to detect subclinical mastitis caused by intramammary infections. In dairy goats, SCC is greatly influenced by non-infectious factors, which complicates the interpretation. The aim of this research paper was to determine the association between SCC, intramammary infections and non-infectious factors including parity, season, lactation stage, and milk yield in dairy goats. In this longitudinal study, 451 goats from four Norwegian dairy goat herds were sampled for bacteriology and SCC up to nine times during two lactations. Factors like parity, milk yield, and stage of lactation were retrieved from the Norwegian goat recording system.

**Results:**

The most prevalent udder pathogen findings were *Staphylococcus caprae* (6.8%), *Staphylococcus warneri* (6.3%), and *Staphylococcus epidermidis* (3.8%), all of which had a mild but significant impact on SCC. *Staphylococcus aureus* was detected in 3.6% of the udder halves and had a major effect on SCC. Parity, stage of lactation, season, and milk yield significantly influenced SCC.

**Conclusions:**

This study highlights that intramammary infections caused by *Staphylococcus aureus*, along with factors such as increasing parity and the seasonal effects of pasturing, significantly influence the SCC. Understanding these key contributors is essential for improving udder health management and improving milk quality in goat milk production.

**Supplementary Information:**

The online version contains supplementary material available at 10.1186/s12917-024-04348-6.

## Background

Mastitis, primarily caused by bacterial intramammary infections (IMI), significantly impacts economic losses and antimicrobial usage in milk production. Due to its often subclinical nature, inflammation indicators, particularly somatic cell count (SCC), are commonly employed for screening [1]. Whilst IMI are the main source of variation in SCC in dairy cows [1–3], this is probably not true for dairy goats, where a higher proportion of variation in SCC is of non-infectious origin [4, 5]. Non-infectious factors influencing SCC in goats include e.g. lactation stage, breed, parity, and various stressors [6–8], but the amount of variation explained by these different factors is largely unexplored.

The literature on the cell composition in healthy compared with infected goat udders is limited. For animals free of IMI, goat milk contains a higher proportion of neutrophils than cow’s milk [9–11]. Furthermore, goat milk contains a higher number of non-leukocytic epithelial cells and apocrine particles [12, 13]. However, most of the apocrine particles lack nucleus, and should therefore not be counted with modern counting methods [10, 13].

Due to the large variation of non-infectious origins, SCC is questioned as an appropriate indicator of udder health and milk quality in dairy goats [5, 14]. Nevertheless, SCC is widely used for udder health surveillance in dairy goats, since few cost-efficient alternatives are available [15, 16]. In Norway, a threshold of 1 million cells/mL has been used as a guideline for suspecting IMI at goat level [17]. Several dairy companies, including TINE in Norway, include bulk milk SCC as one of the payment parameters also in goat milk [9, 17]. To account for the marked seasonal variation of bulk milk SCC in goat milk in Norway, the cutoff for premium payment was 1,2 million cells per mL in 2022, calculated as a 12-month geometric mean.

Staphylococci are the predominant cause of IMI in dairy goats [18]. *Staphylococcus (S.) aureus* is recognized as the most important udder pathogen due to its ability to cause clinical mastitis and persistent infections with potential for contagious spread [19, 20]. The role of non-aureus staphylococci and mammaliicocci (NASM) in udder health is more unclear. Although the SCC response is milder than for IMI caused by *S. aureus* [21, 22], some studies have shown the ability of NASM to cause persistent infections, also across the dry period [23]. The prevalence in a herd is often relatively high, which may in sum influence the bulk milk SCC [18, 24].

The dairy goat population in Norway consists of 239 herds with an average herd size of 138 lactating goats [25]. The Norwegian dairy goat is the main breed. All Norwegian dairy goat herds participated in the eradication programme “Healthier goats”, where Caprine Arthritis Encephalitis (CAE), caseous lymphadenitis (CLA), and paratuberculosis were eradicated from the Norwegian goat population [26]. The total annual milk production is approximately 18 million litres [25]. The goat milk production in Norway is seasonal, and most farms have concentrated kidding from December to March, and with the goats on pasture between June and September. Pasture-based milk production is an important goal, and approximately 30% of the goat milk is produced on pasture [25]. However, the bulk milk SCC increases when the goats are let out on pasture [27], and this, as well as other management-related factors which contribute to the SCC in goats, need to be explored further.

A better understanding of the factors influencing goat milk SCC could potentially increase its value in the udder health work and provide insights for developing appropriate quality payment systems for goat milk. Thus, the objective of this study was to determine the association between SCC, intramammary infections and non-infectious factors in dairy goats.

## Results

### Descriptive statistics

Descriptive data of the four included herds are provided in Table 1. The VaDia milking-time testing demonstrated appropriate milking machine functioning on all four farms at the end of the study.

**Table 1:** Description of the four Norwegian dairy goat farms (A-D) included in the study

		Farm		
Description	A	B	C	D
Bulk milk somatic cell count^a^				
2020	1151	1238	753	831
2021	888	1270	610	572
2022	806	1326	598	864
Bulk milk somatic cell count^b^				
Indoor spring	499	911	264	540
Pasture	1554	1673	1321	1312
Fall	1189	1579	659	1170
Herd size^c^	92	119	115	62
Average annual milk production^d^	750	509	741	688
Proportion of goats > third parity (%)	34	40	63	25
Replacement rate (%)				
2021	31	20	22^e^	41
2022	21	23	16^e^	25
Flooring/bedding	Metal mesh	Plastic mesh	Deep bedded straw	Plastic mesh
Milking system	Pipeline	Parlour	Parlour	Parlour
Automatic cluster removal	No	Yes	Yes^f^	No
Access to outdoor areas outside pasture season	No	No	Yes	Yes
Milking-time testing^g^				
Machine on time^h^	01:47	02:08	01:37	01:39
Overmilking^i^	00:07	00:12	00:04	00:47
Average vacuum level, kPa^j^	35.04	34.66	34.23	35.61

^a^12-month geometric mean (*1000 cells/mL)

^b^Mean bulk milk somatic cell count (*1000 cells/mL) according to seasons (mean of 2021 and 2022)

^c^Average annual number of milk goats

^d^Kg milk pr goat > 1 parity, 280 days lactation

^e^Farm C did not have a constant replacement rate the years before the study

^f^Automatic cluster removal at the home farm only

^g^Mountain farm C, home farm A, B and D

^h^Time (minutes, seconds) from start milking to end milking

^i^Time (minutes, seconds) in the overmilking period (no milk flow but still milking)

^j^kPa in the short milk tube (SMT) during main milking (b-phase)

**Fig. 1:**
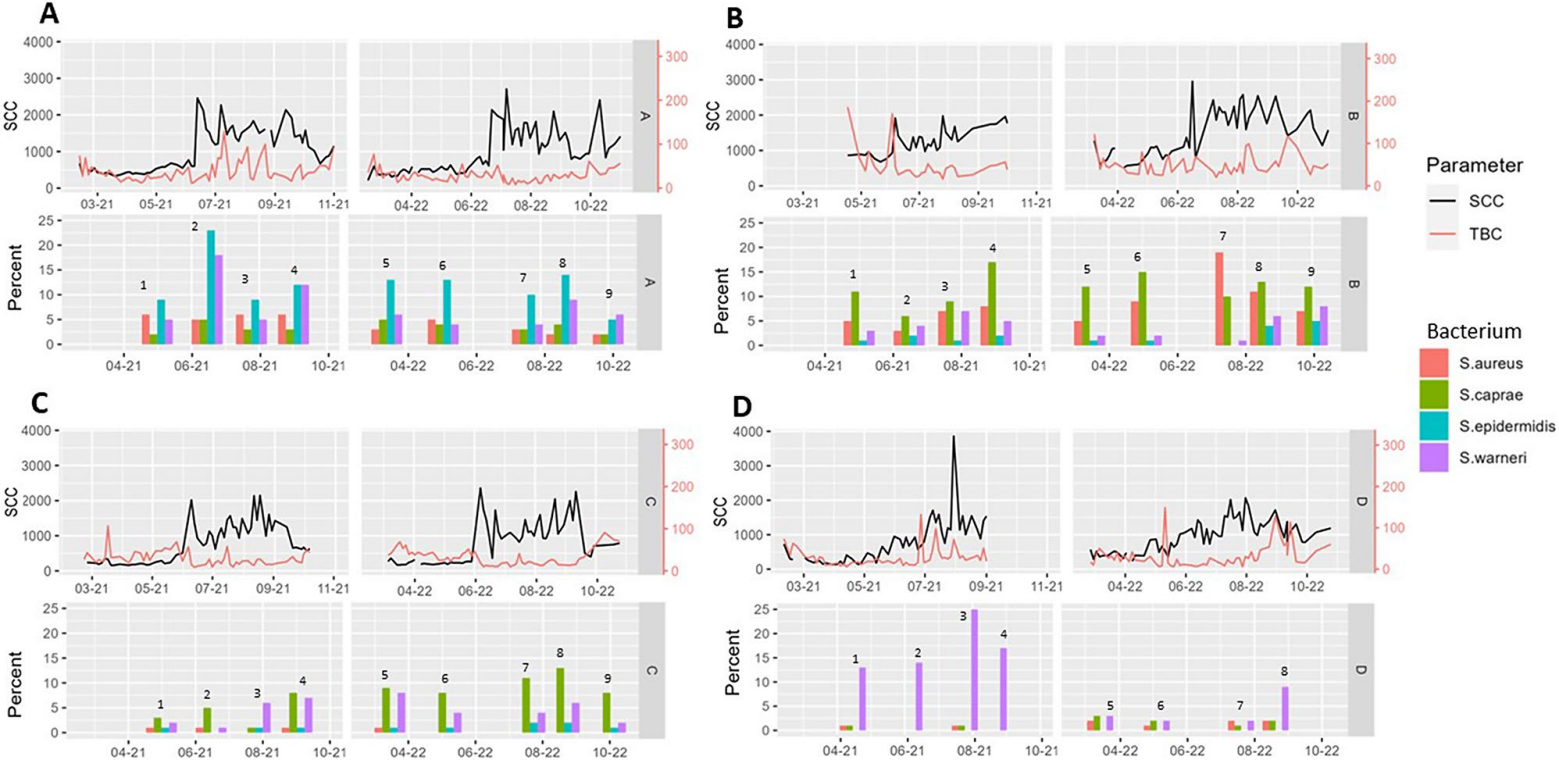
Bulk milk somatic cell count (SCC) x 1000 cells/mL and total bacterial count (TBC) x 1000 cells/mL in four Norwegian dairy goat herds (A-D) in 2021 and 2022, combined with the percentage of infected udder halves of the most frequently detected udder pathogens (*Staphylococcus aureus (S. aureus)*,* Staphylococcus caprae (S. caprae)*,* Staphylococcus epidermidis (S. epidermidis)*,* Staphylococcus warneri (S. warneri*), at the up to nine sampling events (1–9)

Plots of the bulk milk somatic cell count and total bacterial count, as well as the herd prevalence (percent infected udder halves) in each sampling event are shown in Fig. 1. The marked increase in bulk milk SCC in farms A, B, and C in June both years (Fig. 1) occurred at the first milk delivery after the herds were released to pasture. The SCC-profiles showed a farm-specific pattern that was relatively consistent over the two years (Fig. 1). The most common bacterial findings (at udder half level) were *S. caprae* (6.8%), *S. warneri* (6.3%), *S. epidermidis* (3.8%), and *S. aureus* (3.6%). The distribution of samples and number of goats according to IMI-status at goat level is presented in Table 2. The percentage of infected udder halves showed little seasonal variation (Fig. 1). The IMI-status was relatively evenly distributed according to parity (Additional file 1, Table A1).

**Table 2:** Udder half bacteriology results aggregated to a goat-level category with the intramammary infection status (IMI-status) in 3242 samples obtained from 451 goats on four farms (A-D) in 2021 and 2022

	Farm				
	A	B	C	D	Total
IMI-status	Number of samples (number of goats)				
Negative	466 (99)	390 (115)	715 (126)	386 (80)	1957 (391)
* S. aureus* ^a^	71 (29)	133 (58)	5 (5)	15 (7)	224 (99)
* S. epidermidis* ^a^	111 (41)	17 (11)	16 (5)	0 (0)	144 (57)
* S. caprae* ^a^	22 (8)	119 (53)	130 (47)	11 (7)	282 (112)
* S. warneri* ^a^	65 (33)	35 (25)	49 (37)	73 (33)	222 (125)
Other^b^	93 (54)	80 (46)	101 (51)	44 (23)	318 (169)
Contamination	17 (16)	49 (39)	29 (27)	1 (1)	95 (81)

^a^ *Staphylocuccus is abbreviated S.*

^b^Other: This category includes mixed NASM infections, as well as other bacteria identified (n = number of udder halves with this bacterial finding): Other non-aureus staphylococci and mammaliicocci (*n* = 103), *Pseudomonas* sp (*n* = 29) *Trueperella pyogenes* (*n* = 1), *Streptococcus uberis* (*n* = 1), alpha streptococci (*n* = 8))

### Statistical models

The dataset included altogether 3242 observations. Twenty-two observations with missing values in either bacteriological culture, milk yield or SCC measurements were excluded. Ninety-five observations were excluded due to contamination, resulting in 3125 observations from 451 goats used in the multivariable model including all observations. Description of lnSCC by the categorical variables included in the models (main effects) is provided in Additional file 1 (Table A2).

Model estimates including all observations are provided in Table 3. Intramammary infections caused by *S. aureus*, increasing parity, and the pasture season were the categories with highest impact on SCC (Table 3). A significant negative interaction was seen between parity and IMI caused by *S. aureus*, *S. epidermidis* and *S. warneri*, meaning that these infections have a stronger effect on SCC in first parity goats than in the older goats. The model explained 57% of the variation in SCC (conditional R^2^). The approximate contribution of the fixed effects to the variation in lnSCC was 15% for sampling period, 12% for parity, 7% for IMI-status, 3% for milk yield and 3% for year. Altogether, the non-infectious factors (sum of the contributions of sampling period, parity, milk yield and year) explained 34% of the variation in SCC, whilst infectious factors explained only 7% (IMI-status). The random effect of goat nested within herd explained 16% of the variation in SCC.

**Table 3:** The estimated coefficients (β) and 95% confidence interval (CI) for associations between ln-transformed somatic cell count (lnSCC), intramammary infection status^a^ and non-infectious factors (fixed effects). Based on 3125 samples from 451 Norwegian dairy goats in four herds. Goat nested within herd was included as random effect

Fixed effects	β	CI	*p*
(Intercept)	5.38	5.20–5.56	< 0.001
IMI-status^a^			
Negative	Reference		
* Staphylococcus aureus*	1.60	1.22–1.97	< 0.001
* Staphylococcus epidermidis*	0.90	0.46–1.34	< 0.001
* Staphylococcus caprae*	0.41	0.08–0.73	0.015
* Staphylococcus warneri*	0.50	0.23–0.78	< 0.001
Other	0.10	-0.16–0.36	0.438
Sampling period^b^			
Indoor spring/early lactation	Reference		
Pasture/mid lactation	1.29	1.21–1.37	< 0.001
Fall/late lactation	1.10	1.02–1.19	< 0.001
Parity			
First	Reference		
Second	0.78	0.63–0.93	< 0.001
Third	1.53	1.34–1.72	< 0.001
≥Fourth	1.39	1.23–1.55	< 0.001
Milk yield^c^	-0.30	-0.36 – -0.25	< 0.001
Year			
2021	Reference		
2022	-0.34	-0.42 – -0.27	< 0.001
Interaction terms			
IMI [S.aureus] * Parity [Second]	-0.33	-0.79–0.14	0.171
IMI [S.epidermidis] * Parity [Second]	-0.56	-1.09 – -0.04	0.036
IMI [S.caprae] * Parity [Second]	0.03	-0.36–0.43	0.868
IMI [S.warneri] * Parity [Second]	-0.11	-0.50–0.28	0.595
IMI [Other] * Parity [Second]	0.54	0.19–0.88	0.002
IMI [S.aureus] * Parity [Third]	-0.25	-0.82–0.32	0.387
IMI [S.epidermidis] * Parity [Third]	-0.80	-1.54 – -0.06	0.034
IMI [S.caprae] * Parity [Third]	-0.49	-0.99–0.02	0.059
IMI [S.warneri] * Parity [Third]	-0.55	-1.01 – -0.10	0.016
IMI [Other] * Parity [Third]	-0.23	-0.63–0.17	0.256
IMI [S.aureus] * Parity [≥ Fourth]	-0.58	-1.03 – -0.13	0.011
IMI [S.epidermidis] * Parity [> third]	-0.76	-1.29 – -0.24	0.005
IMI [S.caprae] * Parity [≥ Fourth]	-0.31	-0.70–0.08	0.124
IMI [S.warneri] * Parity [≥ Fourth]	-0.54	-0.90 – -0.19	0.003
IMI [Other] * Parity [≥ Fourth]	0.08	-0.23–0.38	0.622

^b^Early lactation: less than 100 days in milk, mid lactation: 100–180 days in milk, late lactation: >180 days in milk

^c^Kg milk at the day of recording

In the model including only culture negative goats (“IMI-free model”), 1901 observations from 384 goats were included (Table 4). The model estimates confirmed a strong effect of season and increasing parity also in goats without IMI, with approximately 19% of the variation explained by sampling period and 17% by parity. There was a significant interaction between parity and sampling period, where SCC increased more in goats in third parity or higher on pasture (Table 4). Overall, the model explained 61% of the variation in SCC (conditional R^2^), whereof 45% of the variation was explained by the fixed effects (marginal R^2^). For both models, plots of the residuals showed that they were approximately normally distributed.

**Table 4:** The estimated coefficients (β) and 95% confidence interval (CI) for associations between ln-transformed somatic cell count (lnSCC), sampling period, parity, milk yield and year. Based on 1901 samples from 384 Norwegian dairy goats in four herds, all of which were culture-negative at the time of sampling. Goat nested within herd was included as random effect

Fixed effects	β	CI	*p*
(Intercept)	5.74	5.51–5.98	< 0.001
Sampling period^a^			
Indoor spring/early lactation	Reference		
Pasture/mid lactation	0.85	0.65–1.05	< 0.001
Fall/late lactation	0.97	0.76–1.19	< 0.001
Parity			
First	Reference		
Second	0.66	0.43–0.89	< 0.001
Third	1.23	0.93–1.53	< 0.001
≥Fourth	1.07	0.83–1.30	< 0.001
Milk yield^b^	-0.38	-0.46 – -0.31	< 0.001
Year			
2021	Reference		
2022	-0.43	-0.52 – -0.34	< 0.001
Interaction terms			
Period [Pasture] * Parity [Second]	0.58	0.29–0.88	< 0.001
Period [Fall] * Parity [Second]	-0.03	-0.34–0.28	0.839
Period [Pasture] * Parity [Third]	0.60	0.24–0.95	0.001
Period [Fall] * Parity [Third]	0.39	0.02–0.77	0.040
Period [Pasture] * Parity [≥ Fourth]	0.90	0.66–1.15	< 0.001
Period [Fall] * Parity [≥ Fourth]	0.31	0.05–0.57	0.021

^a^Early lactation: < 100 days in milk, mid lactation: 100–180 days in milk, late lactation: >180 days in milk

^b^Kg milk at the day of recording

The least square means from the model including all observations are presented back transformed to SCC in Table 5 and provide the expected levels of SCC according to infection status, parity and sampling period.

**Table 5:** Least square back transformed to SCC, estimated from main effects of a mixed model with ln-transformed somatic cell count (lnSCC) as outcome, and intramammary infection status^a^ and non-infectious factors as fixed effects. Based on 3125 samples from 451 Norwegian dairy goats in four herds

Fixed effects	Least square means	95% CI
(Intercept)	217	181–260
IMI-status^a^		
Negative	Reference	
* Staphylococcus aureus*	1075	735–1556
* Staphylococcus epidermidis*	534	343–828
* Staphylococcus caprae*	327	235–450
* Staphylococcus warneri*	357	273–473
Parity		
First	Reference	
Second	473	407–550
Third	1002	828–1212
≥Fourth	871	742–1022
Sampling period^b^		
Indoor spring (early lact)	Reference	
Pasture (mid lactation)	788	728–854
Fall (late lactation)	652	601–713

^b^Early lactation: less than 100 days in milk, mid lactation: 100–180 days in milk, late lactation: >180 days in milk

## Discussion

This study investigated and quantified the relationship between SCC in goat milk, non-infectious factors and intramammary infections in four dairy goat herds in eastern Norway. All lactating goats were sampled for SCC and bacteriological status during nine sampling events over two lactations. The findings reveal that non-infectious factors, such as season, lactation stage and parity, have a major impact on SCC in goats. These findings differ from those reported in dairy cows [1, 2] and indicate that adjustments for non-infectious factors are essential for effectively using SCC to detect IMI in goats.

The farms were selected based on previously high versus low bulk milk SCC to include herds with different herd udder health status. The difference in prevalence of IMI in the herds, however, was not very evident. The two farms selected based on high bulk milk SCC (A and B) had the highest prevalence of *S. aureus*, but the percentage of udder halves with *S. aureus* IMI was at a moderate level, usually between 5 and 10% (Fig. 1). The model estimates confirmed the major effect of *S. aureus* on SCC, suggesting that *S. aureus* control is essential in maintaining good milk quality in dairy goats.

Staphylococci belonging to NASM were the most prevalent findings, with a mild but significant impact on SCC. The most prevalent NASM, *S. epidermidis* and *S. caprae*, are common findings in other studies [21, 23, 28], whilst *S. warneri* is only exceptionally reported. *S. epidermidis*, associated with a moderate SCC response, was detected in the same udder half over several sampling events (not shown). Given the relatively high prevalence, certain NASM can therefore not be completely ignored with respect to udder health and milk quality issues in dairy goats.

Regarding non-infectious factors, this study confirmed the strong association between parity and SCC in goats, which is also shown in several other studies [e. g. 5, 29]. Paape et al. (2007) suggested to cull goats of higher parities to reduce this problem [4]. The reasons for the parity effect are not clear. Goats of higher parities are more likely to have gone through an IMI, which may cause permanent damage to the udder tissue and a greater immune response [1]. Increased risk of mastitis with higher age is also reported [29], but not found in this study. The prevalence of IMI according to parity was, however, probably influenced by the measures introduced by the farmers to reduce IMI during the study period, in particular culling of goats infected with *S. aureus*. The history of IMI may be one part of the explanation for the parity effect, but a strong parity effect was seen also in herds with excellent udder status and systematical culling of goats with IMI over several years, including herds C and D in this study. Furthermore, the effect of parity was even higher in the model including only culture-negative observations in this study. A possible explanation is increased shedding of exfoliated secretory epithelial cells in older goats [13]. The composition of goat milk, including the proportions of leukocytes, cell-like particles and epithelial cells, is a matter of ongoing research in Norway.

Another major contributor to variation in SCC was whether the goats were indoor or pastured. Farms A, B and C had a marked increase in bulk milk SCC when the herds were turned out to pasture, and for farms A and C, the bulk milk SCC during pasture was more than three times the level during the indoor spring period. A previous study has shown the marked increase of SCC in Norwegian dairy goat herds in connection to the pasture season [27] and hypothesized that this sudden increase in SCC when released to pasture was caused by stress and increased movement rather than an increased prevalence of IMI. The hypothesis of a predominantly non-infectious nature of the SCC when goats are out on pasture is strengthened based on the results from this study, where the prevalence of IMI remained relatively stable in the different seasons (Fig. 1). Farm D was the only farm where the increase was not evident at the time the goats were transported to the mountain farm and released to pasture. One of the reasons for the different response in farm D may be that the goats in this herd had access to outdoor areas also outside the pasture season. Furthermore, this farm had a higher replacement rate of goats, i.e. lower median parity of the herd. The model including only culture negative goats showed a significant interaction between parity and season, with goats of higher parities having a stronger SCC response when turned out on pasture. Hence, a herd with a relatively high replacement rate will have lower SCC during pasture. One study found differences in SCC response to stress according to parity [7]. Another explanation may be that the older goats have an altered udder conformation which may result in more mechanical stress during movement. In this respect, the daily walking distances in the pasture season in Norway may be up to 10 km for goats on mountain pasture.

The milking technique was evaluated once at the end of the study period. Although the milking machine functioning was considered appropriate in all farms at that time, all four farmers had potential for improvement of the milking routines by introducing cleaning of all udders before milking. Furthermore, since we did not evaluate the milking technique during the whole study period, factors related to milking routines and their potential association to SCC cannot be evaluated based on these data.

This study was limited to four herds only, which were selected to reflect typical management practices in Norwegian goat milk production, considering factors such as herd size, seasonal production, and the utilization of mountain pastures. When comparing the bulk milk SCC levels from this study to those reported in a recent analysis of 88 Norwegian dairy goat herds [27], it was found that farms A and D exhibited SCC levels close to the national average. In contrast, farms B and C had SCC levels in the upper and lower range, respectively, of what is expected in Norway. Furthermore, the most prevalent udder pathogens in the four herds of this study were identical to those reported from a recent study including 170 Norwegian goat herds over a 10-year period [17].

We aimed to include herds with good udder health as well as herds with room for improvement of the udder health. Based on the results, farm B would benefit from an increased focus on *S. aureus* control. The bulk milk profile in farm B showed higher bulk milk SCC during the indoor spring period, as well as several episodes of increased bulk milk total bacterial counts. The pattern of routinely collected data of bulk milk SCC and TBC may have a potential as a herd-level indicator of udder health, which is further investigated in the second part of this study. To improve udder health, however, goat-level milk recordings and bacterial diagnostics are still essential.

Our results are most relevant for the Norwegian management and breed. However, there are few longitudinal observational studies performed in dairy goats, and the study provides a valuable contribution to the understanding of the SCC response of goats, which differs significantly from that of cows. During the study period, the farmers were continuously kept informed of the sampling results, and this may have contributed to the reduced SCC in the second year of the study, as seen in the negative coefficients of year in both models (Tables 3 and 4). With known infection status of all goats, goats with chronic *S. aureus* IMI were probably detected and culled earlier than normal. Culling of *S. aureus*-positive goats was the main strategy for all four farms included in the study. Four goats in farm A with *S. aureus* were treated at dry-off, which is part of the recommended practice for management of IMI caused by *S. aureus* in Norway [30]. The reduced SCC between the two years based on milk recordings (model estimates) was not always reflected in reduced bulk milk SCC on individual farms (Table 1). A possible reason is that bulk milk samples were analysed every third day throughout the study period, whilst the milk recordings reflect the SCC at nine sampling events. Furthermore, bulk milk SCC may also be manipulated by withholding the milk from some goats from the tank.

The modelling approach used in the study had some limitations. Goat nested in herd was included as a random effect. Given the repeated measures, and the possibility of persistent infections in the same goat, it is likely that the random effect of goat accounted for some of the infectious contribution. Furthermore, the majority of the goats were uninfected, giving an unbalanced model. The low prevalence of IMI in this study contributed to the low amount of variation explained by IMI. Although we analysed a large number of samples, our model included four farms only. Thus, several farm-level management factors could not, due to the low number of farms, be included in the models. The practical approach of this problem was to include farms with comparable management, however, many factors such as the housing, feeding system, and stocking density differed, which will here be captured in the “goat nested in herd” random effect. As all factors included in the models were at goat-level, the inclusion of farm as a random effect did not improve the models. In future studies with larger sample size, possible management factors which affect SCC could be further investigated.

## Conclusions

This study examined and quantified the effects of both infectious and non-infectious factors, along with their interactions, on somatic cell count (SCC) in goats. It was found that Staphylococci were the primary cause of intramammary infections (IMI). Besides IMI, factors such as parity, pasture status, and stage of lactation significantly contributed to variations in SCC. To effectively use SCC for identifying goats with IMI, it is essential to adjust for these influencing factors. The second part of this study will further investigate adjusted SCC thresholds for monitoring udder health.

## Methods

### Study design

In this longitudinal observational study, four herds located in a mountainous area in the east of Norway were followed through two lactations, with altogether nine sampling events. The number of included herds was limited by the resources in the project. The average lactation length (number of days with dairy delivery) for the four herds was 234 (range 206–263) days. The sampling events were categorized into three sampling periods according to the seasonal production of goat milk in Norway (Table 6). The study period was from March 2021 to October 2022.

**Table 6:** Sampling events (*n* = 9) categorized into three sampling periods according to the seasonal production of goat milk in Norway

	Sampling period		
Year	Indoor spring /Early lactation^a^ (March – May)	Pasture/Mid lactation^b^ (June – August)	Fall/Late lactation^c^ (September – October)
2021	1st	2nd, 3rd	4th
2022	5th, 6th	7th	8th, 9th

^a^Early lactation: less than 100 days in milk

^b^Mid lactation: 100–180 days in milk

^3^Late lactation: >180 days in milk

The four herds (A, B, C, D) were selected based on convenient geographical location and management that was representative of Norwegian goat milk production with respect to herd size, breed, utilization of mountain pasture in the summer months, and traditional kidding season (February to April). The geographical area was in eastern Norway and altogether 19 goat milk farms were located in this area. To ensure a variation in infection status of the herds, the bulk milk SCC (12-month geometric mean) the year before the study was evaluated. Based on the researcher’s expertise, two farmers considered to have high bulk milk SCC (farms A and B, approximately 1,2 million cells/mL) and two farmers considered to have low bulk milk SCC (farms C and D, approximately 750,000 cells/mL), were contacted. The first four farmers contacted were all willing to participate in the study. All four herds were certified free from CAE, CLA and paratuberculosis. The goats were milked twice a day during the study period in a pipeline milking system (Farm A) or parlour (farms B, C and D). In all four herds, teats were washed (reusable cloth, plain water) before milking only if visible dirty, and no teat disinfectant was used. Herd A, C and D moved their herds to a mountain farm during the summer months. Herd A and D was transported approximately 25 km, Herd C was walked 7 km. Herd B was pastured in a mountainous area close to the farm. The pastures were located 900–1200 m above sea level.

Bacteriological results and SCC from sampling event four in herds A, B and C was also included in another study evaluating the test performance of bacteriological culture and SCC for detection of *S. aureus* in late lactation [31].

### Sampling and registrations

At each sampling event, composite goat milk recording samples and udder half samples for bacteriology were collected from all lactating goats.

The farmer collected composite goat milk samples for analysis of SCC and milk yield measurement (milk recording samples) the same day or the day before the visit. In the milk recording samples, 40 mL milk were obtained with milk meters. The milk meters were ICAR-certified in all four farms, but the date of last calibration was unknown. The milk samples for bacteriology were collected by dairy technicians, the research group, and/or the farmer. The milk samples for bacteriology were collected before milking, by discarding the first streams of milk, disinfecting the teat with a cotton swab soaked in 70% alcohol, and milking 5 mL into a sterile tube, one for each udder half.

The farmers’ digital herd management tool (www.medlem.tine.no) was continuously updated with the results from the analyses, including bacteriology, milk recordings and bulk milk analyses, and the results could hence be utilized to manage and improve udder health during the study period.

### Milking-time testing

Milking-time testing was performed using the vacuum logger VaDia (https://www.biocontrol.no/products/vadia/) and the corresponding software (Biocontrol, Rakkestad, Norway) as described in [32]. The milking time test was recorded on 5–9 goats (6–10%) in each herd, and observation of the milking technique was carried out once at the end of the study period on farms A, B, and D, as well as at separate mountain farms for farms C and D.

### Laboratory analyses

#### Individual goat milk

The milk recording samples were conserved with bronopol and analysed for SCC by Bentley FTS/FCM (Bentley Instrument Inc, Chaska, MN, USA) at the TINE raw milk laboratory (Heimdal, Norway).

Udder half milk samples were transported cooled, and frozen (-20 °C) upon arrival at the laboratory. The milk samples were thawed and analysed by bacterial culture according to standard procedures [33], with some modifications. Briefly, 0.01 mL of milk from each udder half were spread on washed 5% cattle blood agar plates with esculin and incubated at 37 °C. Plates were read at 24 and 48 h.

Bacterial findings were reported if they grew in pure culture and with five or more colonies (500 colony forming units [cfu]/mL), except for *S. aureus*, which was reported at ≥ 100 cfu/mL [34]. *S. aureus* was identified by typical colony morphology and a betatoxic haemolysis zone, or (if not typical colonies/haemolysis) with MALDI-ToF (Bruker Daltonics, Bremen, Germany). All other colonies were identified with MALDI-ToF. NASM were reported at species-level for *S. epidermidis*, *S. chromogenes*,* S. simulans*,* S. warneri*,* S. haemolyticus* and *S. caprae.* Otherwise, they were grouped as NASM.

#### Bulk milk

Results of routine bulk milk analyses from each delivery in 2021 and 2022 were provided by the dairy company (TINE) and included in the descriptive results. According to the standard routines in TINE, milk was collected every third day, and bulk milk samples obtained at each milk collection were analysed for chemical composition and SCC by Bentley FTS/FCM (Bentley Instrument Inc, Chaska, MN, USA), and total bacterial count (TBC) by BactoCount IBC (Bentley Instrument Inc.). SCC and TBC values > 9999 (*1000 cells/mL) were truncated to 9999.

### Statistical analyses

#### Descriptive

The bulk milk SCC and TBC in the four herds throughout the two years were described with plots. Bacterial findings were summarized by sampling period at udder half level for each sampling. 

### Definition of goat level intramammary infection status

Since SCC was measured in composite goat samples, udder half bacteriology results were aggregated to a goat level category with the intramammary infection status (IMI-status) for the most frequently detected udder pathogens: Sampling events with *S. aureus* detected in one or both udder halves were classified as “*S. aureus*”. Sampling events were classified as “*S. epidermidis*”, “*S. caprae*” and “*S. warneri*” if they had only this finding (i.e. the other udder half was either negative or had the same NASM-finding). Sampling events with no growth in either udder halves were classified as “negative”. Other findings than the abovementioned, including mixed NASM-infections, e.g. *S. epidermidis* in one udder half, and *S. caprae* in the other udder half, were classified as “other”. Sampling events with at least one udder half yielding mixed growth were classified as “contaminated” and were not included in the models.

### Factors associated with SCC

Somatic cell count was transformed with the natural logarithm (lnSCC) due to the right-skewed distribution, which normalized the data distribution. Two linear mixed models with an unstructured covariance structure were used to explore the factors associated with lnSCC. In both models, a random intercept for goat nested within herd was applied. Models with random intercept for farm was also tried but did not give better fit. To choose between competing models the Akaike information criterion (AIC) and the Bayes information criterion (BIC) were used. Model assumptions and quality of model fit was evaluated by visual inspection of residual plots. The first model included all observations (*n* = 3125) and had the IMI-status as the main predictor of interest.

The final model was as follows:

$$\eqalign{ lnSC{C_{ij}} & = \mu + IM{I_{ij}} + SAMP{L_{ij}} + PARIT{Y_{ij}} + M{Y_{ij}} \cr & \quad + YEA{R_{ij}} + IM{I_{ij}}{\rm{*}}PARIT{Y_{ij}} + {\sigma _{ij}} + \>{ \in _{ij}} \cr}$$

, where $\:{lnSCC}_{ij}$ is the dependent variable, *i* corresponds to the *ith* goat, *j* corresponds to the *jth* herd, $\:\mu\:$ represents the intercept, $\:IMI$ represents the IMI-status, $\:SAMPL$ represents the sampling period including season and stage of lactation, as defined in Table 6, $\:PARITY$ represents first, second, third and ≥ fourth parity, $\:MY$ represents the milk yield in kg milk at the test day, $\:YEAR$ is 2021, 2022, $\:IMI$ * $\:PARITY$ represents the interaction between $\:IMI$ and $\:PARITY$, $\:{\sigma\:}_{ij}$ represents the repeated variation of the ith goat within the jth herd, and $\:\>{ \in _{ij}}$ represents residual error.

The second model was specified with a subset of sampling events with culture negative results (“IMI-free model”, *n* = 1901). Sampling period, parity, milk yield in kg and year (as described above) were independent variables. The interaction term “parity*sampling period” was included.

The goodness of fit of the models was evaluated by calculating the coefficient of determination explained by the fixed effects (marginal R^2^) and the combined effect of the fixed and random effects (conditional R^2^) [35]. The approximate contribution of the fixed effects to the overall fit of the model was assessed by calculating the difference between the marginal R^2^ in the full and the reduced (i.e. excluding the predictor of interest) model. Model assumptions and quality of model fit was evaluated by visual inspection of residual plots.

The data were analysed in R, version 4.1.3 (R Core Team, 2022), using the packages lme4 [36], SjPlot [37] and ggbreak [38].

## Electronic supplementary material

Below is the link to the electronic supplementary material.

Supplementary Material 1: Table A1: Number of goats with different intramammary infection status according to parity.Table A2: Description of ln-transformed somatic cell count (lnSCC) by variables included in the multivariable models.

## Data Availability

The data generated and analyzed in this study have been used by the permission of TINE and MIMIRO who own the data. The data might be available upon reasonable request to the corresponding author.
