# Supplementary material for: Somatic cell count in dairy goats I: association with infectious and non-infectious factors
Source: BMC Vet Res. 2024 Nov 6;20:509. doi: 10.1186/s12917-024-04348-6 (PMC11539421; doi:10.1186/s12917-024-04348-6)
Supplement: Supplementary file 1 — Supplementary Material 1: Table A1: Number of goats with different intramammary infection status according to parity.Table A2: Description of ln-transformed somatic cell count (lnSCC) by variables included in the multivariable models. [file 12917_2024_4348_MOESM1_ESM.pdf]

Somatic cell count in dairy goats I: Association with infectious and non-infectious factors

Marit Smistad, Ragnhild Aabøe Inglingstad, Liv Sølverød, Siv Skeie, and Bjørn Gunnar Hansen

Additional file 1

Table A1: Number of goats with different IMI-status[1] according to parity.

Parity	Negative	*Staphylococcus aureus*	*Staphylococcus epidermidis*	*Staphylococcus caprae*	*Staphylococcus warneri*	Other
First	448	36	30	40	60	62
Second	375	72	46	92	48	94
Third	219	25	12	33	31	49
≥Fourth	865	86	52	105	80	177
Total	1907	219	140	270	219	382
[1]Intramammary infection status: Bacterial findings in udder half milk samples analysed by bacterial culture, aggregated to a goat-level category as defined in materials and methods						

Table A2: Description of ln-transformed somatic cell count (lnSCC) by variables included in the multivariable models

	Mean lnSCC	SD	N observations (n goats)
IMI-status			
Negative	6.06	1.37	1907 (385)
Staphylococcus aureus	7.59	1.06	219 (98)
Staphylococcus epidermidis	6.45	1.04	140 (55)
Staphylococcus caprae	6.37	1.11	270 (109)
Staphylococcus warneri	6.55	1.10	219 (122)
Other	6.53	1.15	382 (207)
Parity			
First	5.69	1.22	676 (162)
Second	6.22	1.28	727 (154)
Third	6.77	1.26	369 (83)
≥Fourth	6.53	1.33	1365 (210)
Sampling period			
Indoor spring (early lactation)	5.39	1.27	1019 (432)
Pasture (mid lactation)	6.81	1.15	1098 (425)
Fall (late lactation)	6.67	1.10	1020 (437)
Year			
2021	6.59	1.29	1502 (381)
2022	6.09	1.34	1635 (259)
